# Role of phytohormones and nanomaterials in enhancing plant tolerance to abiotic stress

**DOI:** 10.3389/fpls.2026.1857914

**Published:** 2026-07-02

**Authors:** Ziming Ma, Qi Wang, Lanjuan Hu

**Affiliations:** 1 Jilin Provincial Engineering Laboratory of Plant Genetic Improvement, College of Plant Science, Jilin University, Changchun, China; 2 Root Biology and Symbiosis, Max Planck Institute of Molecular Plant Physiology, Potsdam, Germany

**Keywords:** abiotic stress, nanomaterials, phytohormones, plant growth and development, stress-related genes

## Abstract

Phytohormones act as key endogenous factors and signaling molecules that mediate abiotic stress responses in plants and are the integration centers of plant responses to environmental stimuli, playing an important role in plant resistance to drought, salt, cold, and other stresses. Stress responses are finely regulated through a complex network of different classes of phytohormone signaling pathways. Many transcription factors are able to regulate the content of endogenous plant hormones by influencing hormone synthesis and metabolic gene and stress-related gene expression, which in turn affects plant growth and development and improves plant tolerance to abiotic stresses. Signaling molecules in plant stress responses, such as abscisic acid, ethylene, gibberellin, jasmonic acid, and salicylic acid. Their roles in orchestrating plant responses to abiotic stresses. With global climate change, abiotic disasters have become increasingly frequent in recent years, severely hindering crop growth and development. Nanomaterials have attracted widespread attention from researchers because they can significantly alleviate abiotic stress in crops caused by factors such as salinity, drought, flooding, and heavy metals. This paper reviews recent research progress on the use of phytohormones and nanomaterials to alleviate abiotic stress in plants and elaborates on their underlying mechanisms of action. In the future, we will focus on investigating the roles of phytohormones and nanomaterials in modulating plant responses to abiotic stress, thereby enhancing plant tolerance to such stresses and increasing crop yields to address food security challenges.

## 1 Introduction

**Figure 1:**
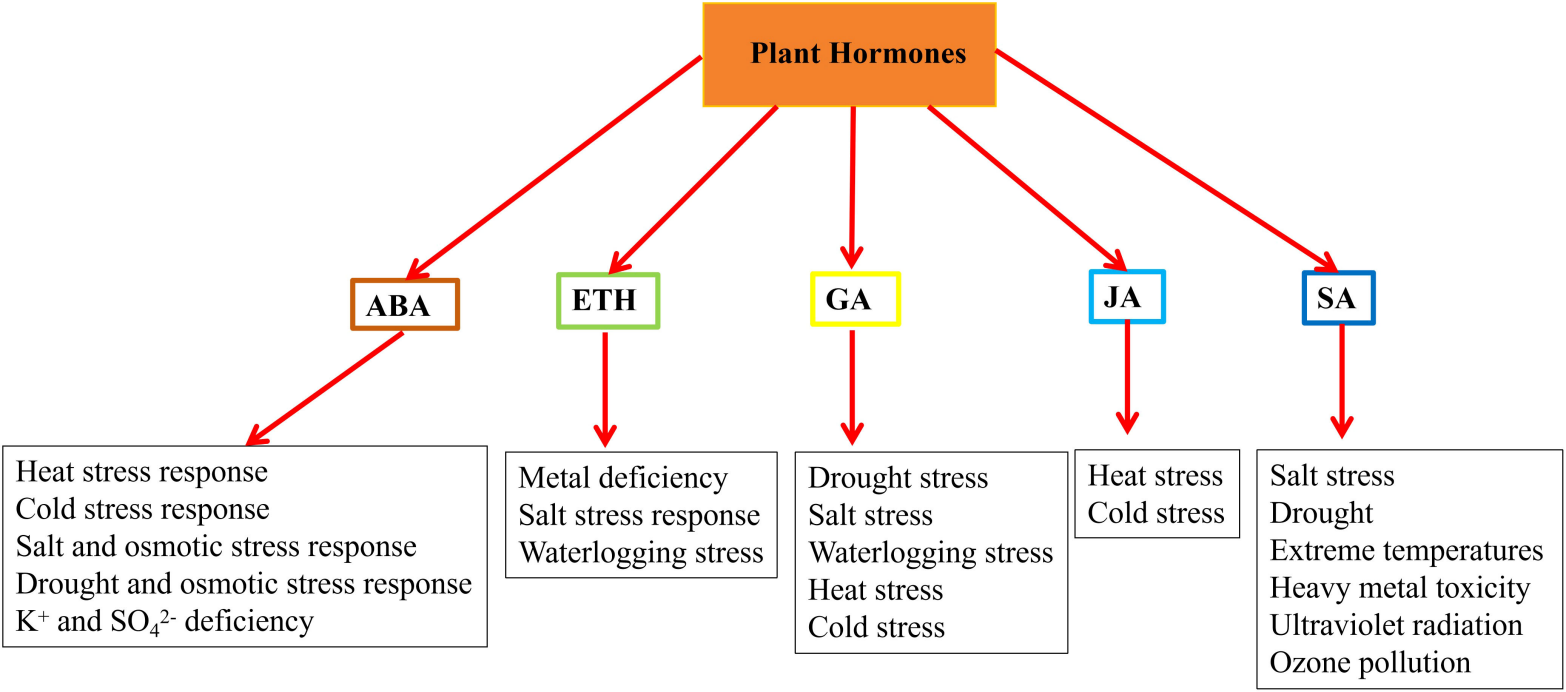
Mechanisms of action of plant hormones in response to abiotic stress. The plant hormone abscisic acid (ABA) can induce: heat stress response, cold stress response, salt and osmotic stress response, drought and osmotic stress response, and K^+^ and SO_4_^2-^ deficiency. The plant hormone ethylene (ETH) can induce: metal deficiency, salt stress response, and waterlogging stress. The plant hormone gibberellin (GA) can induce: drought stress, salt stress, waterlogging stress, heat stress, and cold stress. The plant hormone jasmonic acid (JA) can induce: Heat stress and cold stress. The plant hormone salicylic acid (SA) can induce: salt stress, drought, extreme temperatures, heavy metal toxicity, ultraviolet radiation, and ozone pollution.

Phytohormones are organic signaling molecules produced by plants through their own metabolism that can produce significant physiological effects at very low concentrations. Phytohormones can either function at the site of synthesis or be transported via the vascular system to act in tissues that are relatively distant from the site of synthesis. Phytohormones as signaling molecules have been studied in plant abiotic and biotic stress responses, such as abscisic acid, auxin, brassinosteroids, ethylene, gibberellin, cytokinins, jasmonic acid, strigolactone, and salicylic acid (Bari and Jones, 2009**;** Nakashima and Yamaguchi-Shinozaki, 2013**;** Lata and Prasad, 2011**;** Navarro et al., 2008**;** Nishiyama et al., 2013**;** Verma et al., 2016). These phytohormones not only help plants to resist abiotic and biotic stresses but also regulate plant growth and development. They are independent of each other but also synergistically regulate the developmental processes such as seed germination, nutrient growth, reproductive growth, embryonic development, seed maturation, and dormancy, as well as the adaptation to biotic and abiotic environmental stresses during the growth cycle of plants (Ma et al., 2020**;** Ma et al., 2022**;** Ma et al., 2025**;** Waadt et al., 2022**;** Chakraborty et al., 2025**;** Zhong et al., 2025). Studies have shown that many families of transcription factors are able to hormonally regulate the ability of plants to cope under abiotic stresses (Huang et al., 2021**;** Ma and Hu, 2023**;** Ma and Hu, 2024a**;** Ma et al., 2024b). Because of the complex interactions of different phytohormones and their ability to control a wide range of physiological processes, they can serve as key endogenous factors that mediate plant stress responses. Moreover, since plant hormones are involved in defense responses, their complex intertwined signaling pathways make the generation of fine and efficient stress responses easier (Navarro et al., 2008**;** Nishiyama et al., 2013**;** Verma et al., 2016). Studies have shown that abscisic acid, ethylene, gibberellin, jasmonic acid, and salicylic acid play important roles in orchestrating plant responses to abiotic stresses (Pérez-Llorca et al., 2023**;** Xie et al., 2019**;** Yang et al., 2022) (**Figure 1**). Abscisic acid is the main phytohormone that regulates the response of plants to abiotic stresses, such as drought, salt, cold, heat, and other abiotic stresses that can alter abscisic acid levels (Bari and Jones, 2009**;** Nakashima and Yamaguchi-Shinozaki, 2013). Ethylene is a key plant hormone that plays a complex role in plant responses to abiotic stress, both enhancing plant resistance and, under certain conditions, leading to aging and death. It plays a vital role in defending against adversities such as drought, high salinity, and low temperatures (Bianchetti et al., 2024). Under abiotic stress, gibberellin helps plants adapt to environmental pressures such as drought, salt, and heat tolerance by regulating its own biosynthesis and signal transduction. Its specific functions include controlling leaf elongation, promoting seed germination, flowering, and influencing plant responses to gravity sensing (Khan et al., 2023a). The role of jasmonic acid in abiotic stress primarily involves regulating plant growth and development while acting as a signaling molecule to activate defense responses. When plants encounter drought, salinity, high temperatures, or low temperatures, the jasmonic acid signaling pathway is activated, thereby influencing plant growth and simultaneously inducing the plant to produce a series of defense mechanisms (Wang et al., 2020). Salicylic acid plays a crucial role in plant defense against abiotic stresses by regulating metabolic processes, such as enhancing resistance and inducing defense responses (Nadarajah et al., 2021). Therefore, hormones play a crucial role in plant responses to abiotic stress.

Plant nanobiology is a cutting-edge interdisciplinary subject that has emerged recently. Nanomaterials have a unique small size effect and can enter the interior of plants as nutrient carriers or regulators and participate in plant metabolism, which can promote plant growth and development. Nanozymes are nanomaterials that have catalytic activity similar to natural enzymes (Zhao et al., 2020**;** Zhao et al., 2022a**;** Zhou et al., 2021). In 2007, researchers made a breakthrough discovery confirming that Fe_3_O_4_ nanoparticles are not only superparamagnetic but also exhibit activity similar to that of natural horseradish peroxidase (HRP) (Gao et al., 2007). They have demonstrated that inorganic materials can exhibit enzymatic properties at the nanoscale. Similar to natural enzymes, nanoenzymes can efficiently catalyze enzyme substrates under mild physiological conditions, producing the same reaction products as natural enzymes. They can serve as alternatives to enzymes to regulate cellular metabolism and be used for disease diagnosis and treatment (Yang et al., 2023).

Nanomaterials are materials that have at least one dimension in the range of 1–100 nm in three dimensions and have special physicochemical properties in contrast to the corresponding non-nanomaterials (Arora et al., 2022**;** Auffan et al., 2009). Since the early 1990s, nanotechnology has been rapidly developing in the fields of medicine, energy, and food processing and is widely used in biosensors, water purification, photocatalysis, and antimicrobial agents. In the field of agriculture, nanomaterials can enter into crop plants through their organs such as seeds, roots, and leaves to regulate their physiological and biochemical metabolisms in order to improve their tolerance to biotic and abiotic stresses, and they can also be applied in agricultural production in the form of pesticides, fertilizers, nanocomposites, and nanosensors, which can significantly improve the yield and quality of crops. And the different structures, shapes, sizes, and concentrations of nanomaterials present different effects on crops at different growth periods (Pramanik et al., 2023**;** Paulami et al., 2023**;** Shaw and Honeychurch, 2022). Gao et al. reported that nano-Fe_3_O_4_ has the enzymatic properties of HRP, which can rapidly catalyze the decomposition of H_2_O_2_, and the reaction follows enzymatic kinetics (Gao et al., 2007). Compared with traditional enzyme catalysts, nanoenzymes have the advantages of high catalytic activity, low cost, and easy scalability in production. At the same time, nanoenzymes are mostly composed of inorganic materials, which can ensure that their chemical structure and properties do not change under extreme conditions, they have higher stability than natural enzymes (Huang et al., 2019**;** Zhang et al., 2021). Some studies have shown that nanoenzymes can effectively regulate plant metabolism and significantly improve plant resistance to abiotic stresses and can also promote plant growth (Gao et al., 2007**;** Xu et al., 2025**;** Imtiaz et al., 2023**;** Adeleke et al., 2022**;** Khalid et al., 2023**).** Nanoenzymes can function as antioxidant-like enzymes, which play a role in scavenging reactive oxygen species (ROS) by catalytically converting excess oxygen radicals or hydrogen peroxide produced in plants due to abiotic stresses into oxygen and water (Wu et al., 2017**;** Khan et al., 2022a). Nanoenzymes also have the functions of up-regulating protein expression levels in crops, complexing heavy metal ions, and providing trace elements for plant growth, all of which play an important role in improving the abiotic stress resistance of crops (Mittler et al., 2022**;** Banerjee and Roychoudhury, 2021**;** Xu et al., 2021).

Plants surviving in nature are subjected to a variety of abiotic stresses, taking osmotic stress as an example, among which drought stress is one of the serious unfavorable factors affecting plant growth and productivity (Zhang et al., 2023). Under drought stress, plant cells undergo dehydration and shrinkage, resulting in severe disturbances to cellular metabolism. Water deficiency adversely affects key physiological processes, including photosynthesis, respiration, transpiration, and root development, leading to substantial reductions in plant growth and productivity. Under prolonged or severe drought conditions, these physiological functions may become critically impaired, ultimately resulting in plant death (Handayani and Watanabe, 2020). At the same time, drought stress is the most critical factor limiting the normal growth and development of plants, which causes a much higher reduction in agricultural yields than other abiotic stresses (Gupta and Shrestha, 2023). Drought stress can reduce crop yields; the primary mechanisms include reduced absorption of photosynthetically active radiation by the canopy, decreased photosynthetic efficiency, and a lower harvest index (Ndikuryayo et al., 2022). Salinity is a major abiotic stress that severely restricts plant growth, development, and agricultural productivity worldwide. Salt stress disrupts ion homeostasis through the excessive accumulation of toxic ions, particularly Na^+^ and Cl^-^, thereby inducing both ionic toxicity and osmotic stress. These effects compromise water and nutrient uptake, resulting in cellular dehydration, nutrient imbalances, and stomatal closure. Consequently, photosynthesis, metabolic homeostasis, and growth-related processes are markedly impaired, while senescence is accelerated. If stress conditions persist, the cumulative physiological and biochemical damage may ultimately culminate in plant mortality (Wan et al., 2017). At the same time, due to plant transpiration, Na^+^ and Cl^-^ accumulate in the plant body, causing damage to the osmotic pressure balance in the plant body, an imbalance of intracellular redox reactions, an increase in reactive oxygen species, a deepening of the degree of plasma membrane peroxidation, and damage to the cellular structure, thus inhibiting seed germination and seedling growth (Navarro et al., 2008**;** Nishiyama et al., 2013**;** Verma et al., 2016). Nowadays, more and more scientists are focusing on abiotic stresses, and many studies have shown that some transcription factor family genes are involved in plant resistance to abiotic stresses. For example, transcription factors such as AP2, WRKY, MYB, NAC, C2H2-ZFPs, and so on. They are involved in the response of plants to abiotic stresses such as drought, cold, salinity, and heat (Almeida et al., 2017**;;** Napieraj et al., 2020**;** Jin et al., 2021**;** Luo et al., 2022). To achieve sustainable agricultural development, meet the growing global demand for food, achieve stable and increased food production, and promote food security and sustainable development, it is urgent to mitigate the negative impacts of abiotic stress on crops, and the rapid development of nanotechnology in the field of agriculture has brought new ideas to this end.

In summary, abiotic stresses represent major environmental constraints that adversely affect plant growth, development, and crop productivity worldwide. Consequently, elucidating the molecular, physiological, and biochemical mechanisms that govern plant stress responses and tolerance has become a critical research priority, driving extensive efforts to develop stress-resilient crops capable of maintaining productivity under adverse environmental conditions. With the expectation that by improving the ability of plants to withstand abiotic stresses, we can ensure that the food security crisis caused by rapid population growth will be effectively alleviated worldwide and the yields of major food crops will be increased, with the development of molecular biology, it has been gradually recognized that the expression of plant genes can influence the tolerance of plants under abiotic stresses. This article reviews the role of major plant hormones such as abscisic acid, ethylene, gibberellin, jasmonic acid, and salicylic acid in regulating abiotic stress responses. It also summarizes the research progress in nanomaterials’ action mechanisms and regulation for abiotic stress and proposes suggestions for the development prospects of this field in order to provide a reference for the development of new nanomaterials and their application in resisting abiotic stress. It aims to deepen the understanding of the molecular mechanism of plant hormone regulation of stress responses and provide new ideas for the genetic improvement of crop stress tolerance.

## 2 Mechanisms of phytohormones resistance to abiotic stress in plants.

**Figure 2:**
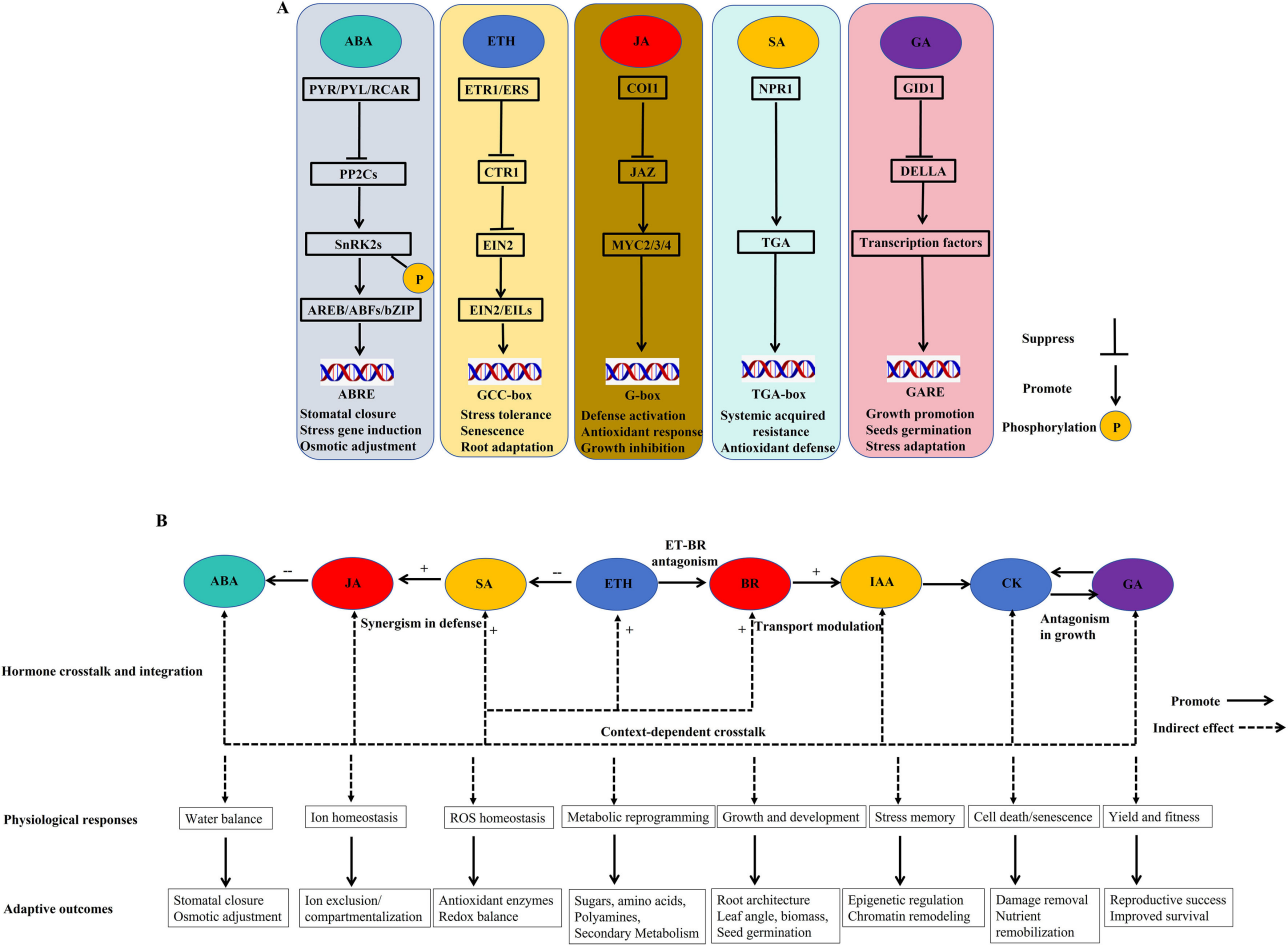
Hormonal signaling networks and their cross-regulatory mechanisms in plant responses to abiotic stress. A: Hormone signaling modules in plants. B: Hormone crosstalk and integration in plants. Under abiotic stress conditions such as drought, salinity, low temperature, high temperature, heavy metal pollution, and flooding, plants perceive environmental changes through membrane receptors, mechanoreceptors, ROS, and Ca²^+^ sensing systems, triggering early signaling events such as ROS bursts, Ca²^+^ influx, MAPK cascades, and phospholipid signaling. Subsequently, hormonal signaling pathways involving abscisic acid (ABA), jasmonic acid (JA), salicylic acid (SA), ethylene (ETH), brassinosteroids (BR), indole acetic acid (IAA), cytokinin (CK), and gibberellin (GA) are activated, regulating the expression of downstream stress-responsive genes through their respective receptor–transcription factor modules. Through synergistic, antagonistic, and context-dependent interactions, phytohormones collectively orchestrate a wide range of physiological and molecular processes, including water balance, ion homeostasis, ROS homeostasis, metabolic reprogramming, growth and developmental regulation, stress memory establishment, and programmed cell death. Among these hormones, ABA serves as a central regulator of osmotic stress responses and stomatal closure, whereas JA and SA often act synergistically to enhance defense signaling. In contrast, ET and BR frequently exhibit antagonistic interactions, while IAA, CK, and GA primarily govern growth- and development-related processes and are tightly integrated with stress-responsive pathways. By coordinately modulating antioxidant defenses, ion transport systems, epigenetic regulation, and nutrient remobilization, these interconnected hormonal networks optimize the trade-off between growth and defense, thereby enhancing stress resilience, physiological fitness, and adaptive capacity. Ultimately, such multilayered hormonal crosstalk enables plants to maintain homeostasis and improve survival and productivity under fluctuating environmental conditions.

Since plants are immobile, they cannot escape abiotic and biotic stresses. Exposure to these stresses throughout their life cycle leads to stunted growth and, in severe cases, death. Consequently, plants have evolved defense responses against multiple stress factors, with responses to specific stresses primarily regulated by relevant plant hormones (Waadt et al., 2022**;** Mittler et al., 2022). In this process, beyond the pivotal role of individual hormone levels, interactions and influences among different plant hormones facilitate the coordinated remedial actions of numerous genes and their regulatory factors during stress responses. Therefore, to gain a deeper understanding of defense response mechanisms, it is particularly urgent to elucidate the intricate connections of cross-talk between various plant hormones (Kurosawa, 1926**;** Rankenberg et al., 2021) (**Figure 2**).

### 2.1 The role of abscisic acid in plant responses to abiotic stress.

Abscisic acid is an important plant hormone known as the “stress hormone”. It accumulates rapidly when plants are exposed to stress conditions such as drought, high salinity, and low temperatures. By promoting stomatal closure to reduce water loss, inhibiting growth, and maintaining bud and seed dormancy, it regulates plant development and enhances stress tolerance (Mukherjee et al., 2023**;** Zha et al., 2025). Salt stress and drought stress among abiotic stresses exert profound effects on plants. These stresses impede water uptake by plant roots, leading to “physiological drought,” while salt directly damages plants, manifesting as stunted growth, leaf damage, and reduced photosynthesis. The combined effects of salt and drought intensify these adverse impacts. Under osmotic conditions caused by high salinity or drought, abscisic acid stimulates stomatal closure, maintains water balance, and regulates the expression of stress response genes (Li et al., 2024**;** Perin et al., 2019**;** Zhu, 2002). Lim et al. found that OsWRKY5 negatively regulates drought tolerance in rice. Its expression is downregulated by drought stress as well as by NaCl, mannitol, and abscisic acid treatments. Loss of *OsWRKY5* activity increases rice sensitivity to abscisic acid, thereby promoting abscisic acid-dependent stomatal closure. OsWRKY5 downregulates the expression of downstream genes of *OsMYB2* in the abscisic acid signaling pathway. As OsWRKY5 acts as a negative regulator of abscisic acid-induced drought tolerance, this strongly suggests that OsWRKY5 may enhance drought tolerance in rice varieties by regulating abscisic acid (Lim et al., 2022). Wei et al. found that rice circadian clock-associated protein 1 OsCCA1 is essential for rice tolerance to salt, osmotic, and drought stress. They identified 692 direct transcriptional targets of OsCCA1, many of which are involved in the abscisic acid signaling pathway. Furthermore, OsCCA1 can directly bind to the promoters of *OsPP108* and *OsbZIP46*, thereby activating their expression. An *Oscca1* knockout mutant generated exhibited increased sensitivity to abscisic acid signaling. In summary, OsCCA1 may confer tolerance to various abiotic stresses in rice by regulating abscisic acid signaling, thereby linking the circadian clock to abscisic acid signaling (Wei et al., 2022).

### 2.2 The role of ethylene in plant responses to abiotic stress.

Ethylene is a gaseous hormone that is widely present in various tissues and organs of plants and plays a role in plant growth and development. It also contributes to plants’ responses to abiotic stress (Van de and de, 2023 Ethylene production and accumulation are finely modulated by endogenous regulatory signals as well as environmental factors, enabling plants to coordinate growth, development, and stress responses. When plants are exposed to biotic or abiotic stressors such as mechanical injury, hypoxia, cold stress, and frost damage, ethylene levels in the plant change. Various stress conditions often lead to an increase in ethylene levels within the plant (Chen et al., 2021a). Djemal et al. found that overexpression of the ethylene-responsive transcription factor *TdSHN1* in durum wheat led to cutin formation and reduced stomatal density. The *TdSHN1*-overexpressing lines exhibited enhanced salt tolerance due to reduced water loss from the leaves (Djemal and Khoudi, 2016). Cheng et al. found that ERF1 may be involved in the salt stress response via the ethylene signaling pathway. In *Arabidopsis thaliana*, *ERF1* expression is significantly induced under both high-salinity and drought stress. Salt stress induction requires both the jasmonic acid and ethylene signaling pathways. *ERF1*-overexpressing lines exhibited enhanced drought and salt tolerance, along with reduced stomatal aperture, resulting in decreased water loss due to transpiration (Cheng et al., 2013). An et al. found that under salt stress, the ethylene-responsive transcription factor MdERF4 is induced and reduces the salt tolerance by binding to and inhibiting the expression of *MdERF3* in apple. They hypothesized that the MdERF4-MdERF3 interaction may serve as a feedback regulatory mechanism for maintaining ethylene homeostasis in plants under salt stress (An et al., 2018). Valluru et al. examined six wheat genotypes that exhibited significant differences in their responses to drought. Under mild drought stress, the above-ground dry weight of the drought-tolerant wheat population was significantly higher than that of the other populations; this phenomenon was significantly correlated with a simultaneous increase in ethylene and abscisic acid levels, as well as an overall decrease in the abscisic acid/ethylene ratio. The *eto1* mutant of *Arabidopsis*, which accumulates higher levels of ethylene *in vivo*, exhibited a slower rate of stomatal closure than control plants when subjected to drought stress. Other studies have suggested that ethylene promotes stomatal closure by positively regulating the production of reactive oxygen species mediated by NADPH oxidase in guard cells (Valluru et al., 2016**;** Desikan et al., 2006**;** Tanaka et al., 2005).

### 2.3 The role of gibberellin in plant responses to abiotic stress.

As a classic plant hormone, gibberellin regulates various processes of plant growth and development, such as promoting stem elongation, leaf expansion, seed germination, flowering, and fruit development (Sandhu et al., 2025). Gibberellin interacts with other hormonal signals within the plant, integrating to form a complex regulatory network that synergistically regulates plant growth and development and helps the plant respond to abiotic stress (Liu et al., 2023). Lu et al. found that two homologous proteins of AtMFT (phosphatidylethanolamine-binding protein), OsMFT1 and OsMFT2, are present in *Oryza sativa*. Under salt stress, seeds from the *Osmft1* mutant germinate faster than those from wild type. Overexpression of *OsMFT1* or *OsMFT2* increases sensitivity to salt stress during seed germination. A transcriptomic comparison of *Osmft1* mutants with wild type under salt-stressed and unstressed conditions revealed multiple differentially expressed genes associated with salt stress, plant hormone metabolism, and signaling pathways. Furthermore, under salt stress, *OsMFT1*-overexpressing seeds exhibited increased sensitivity to gibberellin (Lu et al., 2023). Li et al. identified three WRKY transcription factors OsWRKY24, OsWRKY53, and OsWRKY70 that play roles in abiotic stress and plant hormone responses. They contain two conserved domains, and their promoters harbor multiple cis-regulatory elements that respond to abiotic stress and hormone signals. Under various stress conditions—including darkness, low temperature, salt stress, and drought—as well as following treatment with hormones such as abscisic acid, salicylic acid, jasmonates, and gibberellin, the transcriptional levels of these genes in wild-type seedlings underwent significant changes. The expression level of *OsWRKY24* was downregulated under salt stress, drought, and following treatment with abscisic acid and gibberellin. OsWRKY53 transcripts were induced under darkness, low temperature, salt stress, and drought treatments, while salicylic acid and gibberellin treatments suppressed their expression. Furthermore, the expression level of *OsWRKY70* was upregulated under darkness and low-temperature conditions but was suppressed under salt stress, drought, abscisic acid and gibberellin treatments (Li et al., 2023).

### 2.4 The role of jasmonic acid in plant responses to abiotic stress.

Jasmonic acid hormones are a class of very important lipid-based growth regulators in plants; they play a role in regulating certain key growth and development processes as well as responses to environmental factors, such as the initiation of trichomes on leaf surfaces, anthocyanin accumulation, and responses to freezing stress (Ruan et al., 2019**;** Wang et al., 2021a). Kong et al. identified and characterized the osmotic stress-induced ethylene response factor 15 (PtoERF15), which is involved in regulating the size, density, and cell wall thickness of xylem vessels in *Populus tomentosa* in response to drought stress. Overexpression of *PtoERF15* helps maintain water potential in the stem, thereby enhancing its drought tolerance. PtoERF15 directly regulates *PtoMYC2b*, a key regulator of the jasmonic acid signaling pathway. PtoMYC2b is also involved in the regulation of *Populus tomentosa* vessel morphology. In summary, the PtoERF15-PtoMYC2b transcriptional cascade maintains stem water potential by regulating xylem vessel development, ultimately enhancing the drought tolerance of *Populus tomentosa* (Kong et al., 2023). Wang et al. cloned the *PlWRKY13* gene from peony leaves. Four types of abiotic stress—low temperature, high temperature, waterlogging, and salt stress—all induced the expression of *PlWRKY13*, which was upregulated. Measurements of endogenous hormone levels (jasmonic acid and salicylic acid) revealed that jasmonic acid levels gradually increased following infection with *A. tenuissima*. The overall decrease in the levels of both hormones suggests that they are associated with PlWRKY13 transcription and that PlWRKY13 may be involved in jasmonic acid and salicylic acid mediated disease resistance pathways (Wang et al., 2019a).

### 2.5 The role of salicylic acid in plant responses to abiotic stress.

Salicylic acid is a phenolic hormone. It regulates plant growth and development and also influences photosynthesis, transpiration, and the uptake and transport of ions in plants. Additionally, salicylic acid plays an active role in how plants respond to various abiotic stresses, including cold, drought, salinity, and heavy metals (Nadarajah et al., 2021**;** Prakash et al., 2021). Fan et al. identified 175 differentially expressed genes (DEGs) between the AP2 transcription factor *erf3* mutant and wild type, the upregulated DEGs were primarily enriched in defense-related pathways, including the salicylic acid pathway marker genes *PR2* and *PR5*. Conversely, downregulated DEGs were primarily enriched in pathways responding to wounding/jasmonic acid and abscisic acid/water stress, indicating that ERF3 positively regulates jasmonic acid-mediated wound responses and abscisic acid-mediated abiotic stress responses. Following pathogen infection, ERF3 is induced, thereby suppressing the expression of salicylic acid pathway genes and promoting jasmonic acid-mediated wound responses and abscisic acid-mediated abiotic stress responses (Fan et al., 2025). He et al. identified a NAC transcription factor, GhATAF1, in cotton. This gene is significantly induced by jasmonic acid, salicylic acid, and infection by the bacterial pathogen *Ralstonia solanacearum*. Overexpression of *GhATAF1* enhances the expression of the abscisic acid-responsive gene *GhABI4*, thereby improving the salt tolerance of cotton plants. It also regulates multiple stress response genes, such as *GhAVP1*, *GhRD22*, *GhDREB2A*, *GhLEA3* and *GhLEA6* (He et al., 2016). Wu et al. Identified some WRKY transcription factor genes in *Ipomoea pes-caprae* and 17 highly expressed WRKY genes in the transcriptome under salt stress conditions. The gene *IpWRKY16* was significantly upregulated under salt stress, drought, salicylic acid, and abscisic acid treatments. Under salt stress, sweet potato roots overexpressing *IpWRKY16* exhibited higher superoxide dismutase (SOD), peroxidase (POD), and catalase (CAT) activities, as well as lower malondialdehyde (MDA) content. Using non-invasive microtitration (NMT) technology, significant Na^+^ efflux was observed in the elongation zone of *IpWRKY16*-overexpressing sweet potato adventitious roots. Several ion transporter genes responded to the expression of *IpWRKY16*, with *IbSOS3*, *IbAHA4-1*, and *IbAHA4–2* showing the highest expression levels. Therefore, IpWRKY16 responds to salt stress by regulating these key genes (Wu et al., 2024).

## 3 Nanomaterials and abiotic stress

### 3.1 Mechanisms of nanomaterials’ resistance to abiotic stress in plants.

**Figure 3:**
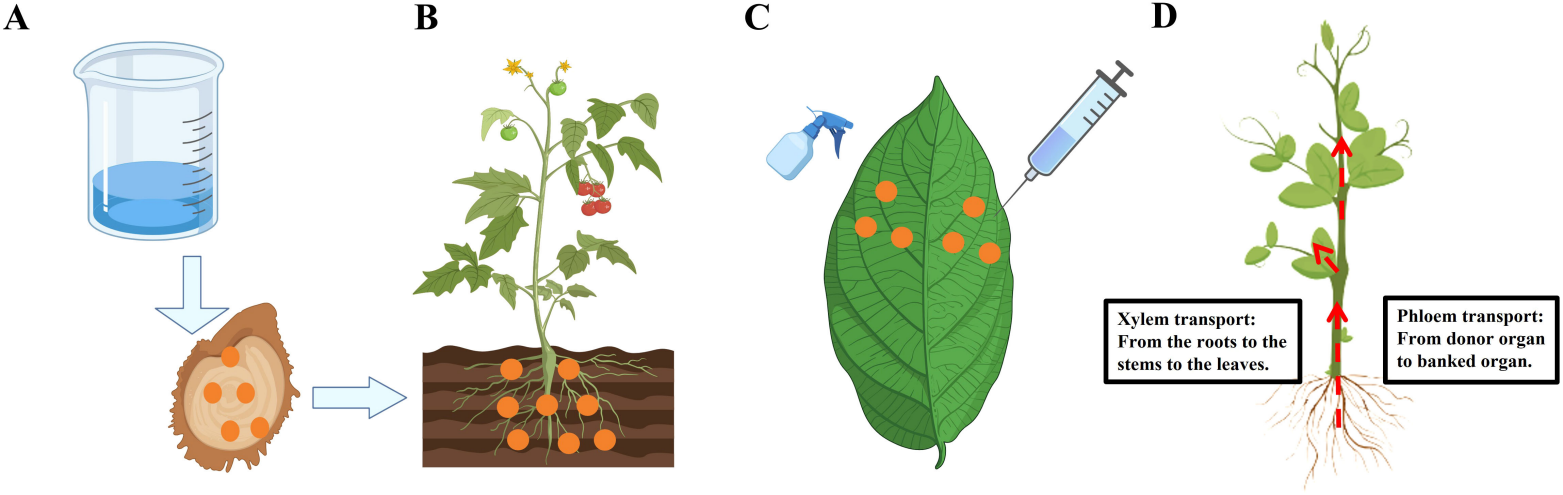
Different pathways of nanomaterial absorption and transportation in plants. **(A)** Seed-induced; **(B)** Root absorption; **(C)** Leaf absorption. **(D)** Two ways of nanomaterial transportation in plants. Nanomaterials are absorbed by plant roots and transported through the xylem and phloem, where they interact with the plant at the cellular, organellar, and molecular levels. This interaction enhances antioxidant capacity, maintains ion homeostasis, improves photosynthesis, and regulates stress signaling networks, thereby increasing the plant’s tolerance to abiotic stresses such as drought, salinity, extreme temperatures, heavy metals, and waterlogging. Ultimately, this promotes plant growth, yield, and productivity. Yellow dots represent nanomaterials. Image materials are generated from https://www.biogdp.com. .

With the increasing frequency and intensity of climate change-related environmental challenges, abiotic stresses such as drought, salinity, extreme temperatures, flooding, and heavy metal contamination have become major constraints on crop productivity and global food security (Šola et al., 2025**;** Eckardt et al., 2023). These stresses disrupt plant growth, development, and physiological homeostasis, thereby necessitating the development of innovative and sustainable approaches to improve crop stress tolerance. Among emerging technologies, nanotechnology has attracted considerable attention owing to its unique potential to enhance plant performance under adverse environmental conditions (Imran et al., 2021**;** Imran, 2024). Nanomaterials can enter plant tissues through seeds, roots, and leaves in a tissue-specific manner (**Figure 3**) (Jiang et al., 2025), where they modulate a range of physiological and biochemical processes. By improving photosynthetic efficiency, enhancing nutrient acquisition, mitigating oxidative and heavy metal-induced damage, and promoting soil health, nanomaterials have emerged as promising tools for strengthening plant resilience and supporting sustainable agricultural production under abiotic stress conditions.

#### 3.1.1 Plants absorb nanomaterials through their root systems

Root uptake represents one of the principal routes through which nanomaterials enter plants and can occur following either soil application or exposure through nutrient solutions. Upon reaching the root epidermis, nanomaterials are internalized primarily via two transport pathways: the apoplastic (extracellular) pathway and the symplastic (intracellular) pathway, enabling their subsequent translocation to different plant tissues. In the apoplastic pathway, nanomaterials move through cell wall pores and diffuse along the extracellular spaces between the cell wall and plasma membrane before encountering selective barriers such as the endodermis (Avellan et al., 2017**;** Dutta et al., 2023**;** Vishwakarma et al., 2023). In contrast, the symplastic pathway involves the crossing of the plasma membrane, followed by intracellular transport through the cytoplasm and cell-to-cell movement via plasmodesmata. Additionally, nanomaterials may gain entry into root tissues through damaged regions of the root system (Khan et al., 2022b**;** Butova et al., 2023). The uptake efficiency of nanomaterials is strongly influenced by their physicochemical properties, including particle size, surface charge, morphology, and surface chemistry. Among these factors, particle size plays a critical role in determining root uptake and translocation. For example, roots of *Nicotiana xanthi* can efficiently absorb 3.5-nm gold nanoparticles (AuNPs), whereas larger 18-nm AuNPs are largely retained on the root surface (Sabo-Attwood et al., 2012). Similarly, *Arabidopsis* roots readily internalize AuNPs smaller than 5 nm but exhibit limited uptake of particles ranging from 7 to 108 nm. Surface charge also significantly affects nanoparticle–root interactions. Negatively charged nanoparticles can more readily penetrate root tissues and migrate through plasmodesmata, whereas positively charged nanoparticles tend to adsorb to root cap mucilage, stimulating further mucilage secretion and thereby restricting their entry into internal root tissues. In addition to nanoparticle characteristics, plant-related factors also influence Nanomaterials uptake. Variations in plant species, developmental stage, and root exudate composition can substantially alter nanoparticle bioavailability, rhizosphere interactions, and uptake efficiency (Stolte and Ristroph, 2024**;** Taylor et al., 2014).

In soil ecosystems, naturally occurring charged nanoparticles can interact electrostatically with soil ions, such as K^+^ and Ca²^+^, thereby enhancing the soil cation exchange capacity and influencing nutrient availability (Guleria et al., 2023). Similarly, the application of engineered nanomaterials in hydroponic systems has been reported to improve nutrient uptake under abiotic stress conditions, increase leaf chlorophyll content, and strengthen plant defense responses (Wang et al., 2019b). Moreover, exogenously applied nanomaterials can modify the physicochemical properties of soils, thereby altering the composition and activity of soil microbial communities. These changes may improve rhizosphere conditions, enhance nutrient cycling, and create a more favorable environment for root growth and development. However, the effects of nanomaterials on soil ecosystems are not universally beneficial and depend strongly on nanoparticle type, concentration, and environmental context. For example, Kulikova et al. reported that the partial dissolution of silver nanoparticles (AgNPs) releases Ag^+^ ions, which promote the oxidation of soil organic matter and the disruption of soil aggregates, resulting in elevated concentrations of metal ions in the soil solution. These changes were associated with significant reductions in water uptake, shoot biomass, and root biomass in wheat plants (Kulikova et al., 2020). Likewise, Liu et al. demonstrated that exposure to 20 μg L^-^¹ AgNPs reduced the abundance of soil microorganisms, inhibited the growth of both *Eisenia foetida* and plants, and disrupted the colonization of beneficial rhizosphere microorganisms. Consequently, nutrient and water acquisition by plants was impaired, leading to reduced plant performance (Liu et al., 2019). Collectively, these findings suggest that while nanomaterials possess considerable potential to improve soil fertility, nutrient utilization, and plant stress tolerance, their environmental impacts require careful evaluation to ensure their safe and sustainable application in agricultural systems.

#### 3.1.2 Plants absorb nanomaterials through their leaves

Foliar application is widely recognized as an effective strategy for delivering nanomaterials into plants and is typically performed through leaf infiltration or foliar spraying. The incorporation of surfactants can enhance the adhesion, retention, and diffusion of nanomaterials suspensions on leaf surfaces, thereby improving uptake efficiency (Wu et al., 2017**;** Hu et al., 2020). Following deposition on the leaf surface, nanomaterials may penetrate the cuticle through either hydrophilic or lipophilic pathways, depending on their physicochemical properties, or enter directly through stomatal openings. Once internalized, nanomaterials are distributed within mesophyll tissues and can undergo long-distance transport via the vascular system, particularly the phloem, facilitating their movement from source leaves to sink tissues such as young shoots, roots, and developing organs (Ali et al., 2021**;** Sembada and Lenggoro, 2024**;** Avellan et al., 2019). These characteristics make foliar application a promising and practical approach for the agricultural deployment of nanomaterials under field conditions.

#### 3.1.3 Mechanisms by which nanomaterials enhance plant tolerance to abiotic stress

**Figure 4:**
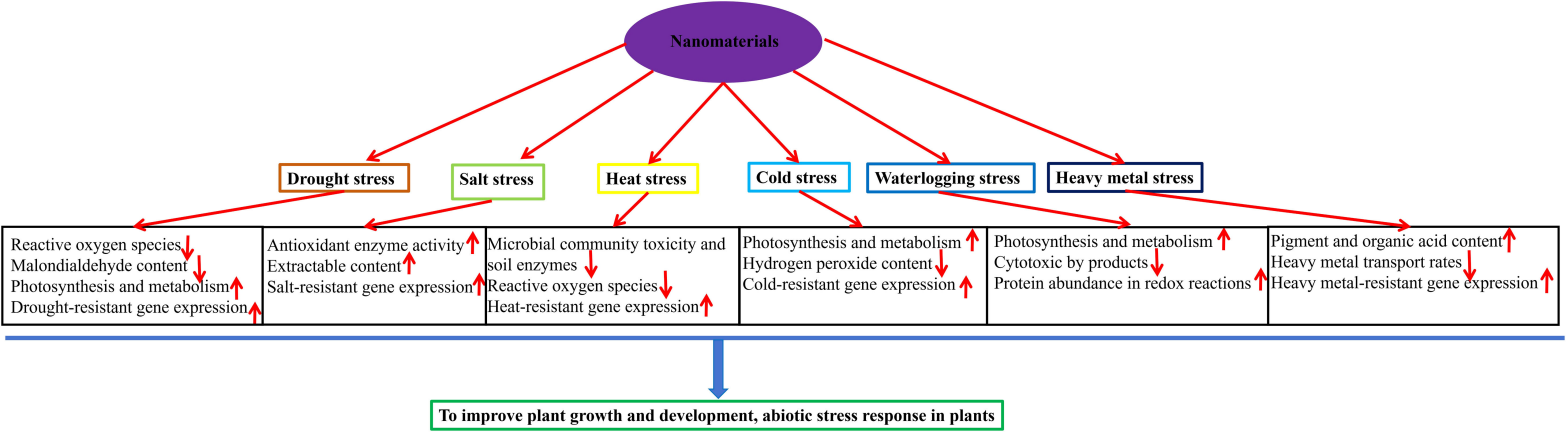
Mechanisms by which nanomaterials enhance crop resistance to abiotic stress. A red up arrow indicates an increase, and a red down arrow indicates a decrease. Drought stress can lead to: a decrease in reactive oxygen species and malondialdehyde content, as well as an increase in photosynthesis, metabolism, and the expression of drought-resistant genes. Salt stress can lead to: an increase in antioxidant enzyme activity, an increase in extractable content, and an increase in salt-resistant gene expression. Heat stress can lead to: a decrease in microbial community toxicity and soil enzymes, a decrease in reactive oxygen species, and an increase in heat-resistant gene expression. Cold stress can lead to: an increase in photosynthesis and metabolism, a decrease in hydrogen peroxide content and an increase in cold-resistant gene expression. Waterlogging stress can lead to: an increase in photosynthesis and metabolism, a decrease in cytotoxic byproducts and an increase in protein abundance in redox reactions. Heavy metal stress can lead to: an increase in pigment and organic acid content, a decrease in heavy metal transport rates and an increase in heavy metal-resistant gene expression.

Nanomaterials function as important regulators of plant–environment interactions by modulating physiological, biochemical, and molecular responses to abiotic stress. Their ability to enhance nutrient acquisition, maintain cellular redox homeostasis, improve photosynthetic efficiency, and activate stress-responsive signaling networks contributes to enhanced stress tolerance and resilience. As a result, plants can more effectively adapt to adverse environmental conditions, thereby sustaining growth, reproductive success, yield, and crop quality under stress conditions (**Figure 4**).

##### 3.1.3.1 The role of nanomaterials under drought stress

Drought stress is one of the most severe environmental constraints affecting plant growth and productivity. Under water-deficit conditions, root systems often undergo structural remodeling characterized by the loss of fine roots and lateral roots, leading to reduced water and nutrient uptake capacity. Consequently, plants experience cellular dehydration, impaired physiological functions, growth inhibition, and, under prolonged stress, eventual mortality (Zhu, 2016). In addition, drought stress disrupts cellular redox homeostasis, resulting in excessive accumulation of ROS, which cause oxidative damage to lipids, proteins, and nucleic acids. Accumulating evidence suggests that nanomaterials can effectively alleviate drought-induced damage through multiple physiological, biochemical, and molecular mechanisms. These include enhancing photosynthetic performance, strengthening antioxidant defense systems, improving water-use efficiency, modulating phytohormone homeostasis, and activating stress-responsive genes (Faraji and Sepehri, 2020**;** Sun et al., 2021**;** Wang et al., 2024). Furthermore, nanomaterials have been shown to improve seed vigor and germination under drought conditions by regulating hormonal balance and maintaining cellular metabolic activity (Wang et al., 2024). Several studies have demonstrated the effectiveness of nanomaterials in enhancing drought tolerance. For instance, Rezayian et al. reported that iron nanoparticles (1.5 and 3 mg L^-^¹) significantly improved the growth and stress resistance of rapeseed plants exposed to polyethylene glycol-induced drought stress. The enhanced tolerance was associated with increased activities of antioxidant enzymes, including catalase and polyphenol oxidase, as well as elevated accumulation of non-enzymatic antioxidants such as phenolics, flavonols, and flavonoids. These responses contributed to improved membrane stability and greater drought resilience (Rezayian et al., 2023). Similarly, foliar application of zinc oxide nanoparticles (ZnO-NPs) at 200 mg Zn L^-^¹ enhanced the activities of catalase, peroxidase, and superoxide dismutase in soybean under drought conditions, resulting in improved physiological performance and higher seed yield compared with conventional ZnSO_4_ treatment (Shirvani-Naghani et al., 2024). In addition to metal-based nanoparticles, carbon-based nanomaterials have also shown considerable potential in drought mitigation. As a nanoscale water-retaining amendment, graphene oxide (GO) enhanced drought tolerance in soybean by increasing antioxidant enzyme activities, modulating phytohormone levels, and upregulating drought-responsive genes, including *GmP5CS*, *GmGOLS*, *GmDREB1*, and *GmNCED1* (Zhao et al., 2022b). These molecular and physiological adjustments improved plant water status and stress adaptation under drought conditions. Collectively, current evidence indicates that nanomaterials enhance plant drought tolerance through integrated regulation of antioxidant defenses, photosynthetic capacity, water relations, hormonal signaling, and stress-responsive gene expression. These coordinated responses contribute to improved plant growth, survival, and productivity under water-limited conditions.

##### 3.1.3.2 The role of nanomaterials under temperature stress

Temperature stress, encompassing both heat stress and cold stress, represents a major environmental constraint that adversely affects plant growth, development, and productivity. Temperature directly influences enzyme activity and metabolic processes; thus, maintaining an optimal thermal environment is essential for normal plant growth. Exposure to low temperatures can impair cellular metabolism, leading to growth retardation, chilling injury, or even freezing-induced mortality, whereas elevated temperatures accelerate transpiration, disrupt photosynthesis, and ultimately reduce crop yield and quality (Ding and Yang, 2022**;** Cvetkovska et al., 2022**;** Guy et al., 2008). In response to temperature stress, excessive accumulation of ROS, membrane instability, and metabolic imbalance frequently occur, resulting in cellular damage and impaired physiological performance.

Recent studies have demonstrated that nanomaterials can alleviate both heat- and cold-induced damage through multiple mechanisms, including the regulation of rhizosphere conditions, enhancement of antioxidant defense systems, maintenance of redox homeostasis, and modulation of stress-responsive gene expression. By mitigating oxidative damage and preserving physiological functions, nanomaterials contribute to improved plant adaptation and resilience under adverse temperature conditions. The beneficial effects of nanomaterials under heat stress have been demonstrated in several crop species. For example, Yadav et al. evaluated the effects of green-synthesized ZnO nanoparticles (30 nm) and conventional ZnSO_4_ fertilizer on rice grown under a Free-Air Temperature Elevation (FATE) system. Compared with untreated plants, soil application of ZnO-NPs significantly enhanced photosynthetic rate and stomatal conductance. Under elevated-temperature conditions, ZnO-NPs also promoted the accumulation of protective metabolites and antioxidant defenses, including increased levels of protein, proline, CAT, and SOD, compared with ZnSO_4_ treatment. Furthermore, ZnO-NPs significantly increased the number of productive tillers, filled grains, and total grain yield, highlighting their potential to improve crop performance under heat stress (Yadav et al., 2026). Nanomaterials have also shown considerable promise in enhancing tolerance to low-temperature stress. Mahmoudi et al. reported that postharvest treatment of plum fruits (*Prunus domestica* L.) with glycine betaine-coated chitosan nanoparticles (CTS-GB-NPs; 5 and 10 g L^-^¹, 150 nm) effectively alleviated chilling injury during cold storage. The nanoparticle coating reduced weight loss and tissue softening, enhanced antioxidant enzyme activities, and maintained ROS homeostasis, thereby improving cold tolerance and preserving fruit quality. Notably, treated fruits retained superior nutritional quality and storage performance even after 40 days at 1 °C, demonstrating the effectiveness of CTS-GB-NPs in extending shelf life and reducing postharvest losses (Mahmoudi et al., 2022). Collectively, these findings indicate that nanomaterials can substantially enhance plant tolerance to temperature stress by strengthening antioxidant defense systems, maintaining cellular redox balance, protecting photosynthetic machinery, and preserving metabolic homeostasis. Through these coordinated effects, nanomaterials improve plant survival, productivity, and product quality under both high- and low-temperature conditions, highlighting their potential for enhancing crop resilience in the face of increasingly variable climatic environments.

##### 3.1.3.3 The role of nanomaterials under salt stress

Salinity is one of the most detrimental abiotic stresses affecting plant growth, development, and agricultural productivity worldwide. Salt stress disrupts cellular homeostasis through osmotic stress, ion toxicity, and nutrient imbalance, ultimately impairing plant physiological and metabolic processes. Excessive salt accumulation in the rhizosphere reduces water availability, leading to physiological drought, while the overaccumulation of toxic ions, particularly Na^+^ and Cl^-^, interferes with nutrient uptake and cellular function. Consequently, photosynthetic efficiency declines, root development is inhibited, leaf necrosis and chlorosis may occur, and severe stress can ultimately result in plant death (Zhao et al., 2021**;** Zhou et al., 2024**;** Yang and Guo, 2018).

Recent studies have demonstrated that nanomaterials can effectively alleviate salt-induced damage through multiple physiological, biochemical, and molecular mechanisms. These include the maintenance of ion homeostasis, enhancement of antioxidant defense systems, regulation of osmotic adjustment, improvement of photosynthetic performance, and modulation of stress-responsive gene expression. Through these coordinated responses, nanomaterials help plants maintain cellular integrity and physiological function under saline conditions. For example, Ghassemi-Golezani et al. evaluated the effects of biochar and biochar-based nanocomposites containing magnesium oxide (BNC-MgO) and manganese oxide (BNC-MnO) on the salt tolerance of Carthamus tinctorius L. The application of these materials significantly increased the accumulation of essential nutrients, including potassium, magnesium, and manganese, while enhancing photosynthetic pigment content, maximum photochemical efficiency (Fv/Fm), relative electron transport rate (RETR), and leaf water status. Simultaneously, the treatments reduced Na^+^ accumulation, ROS production, and oxidative damage, resulting in improved biomass production under salt stress. Among the tested treatments, the combined application of BNC-MgO and BNC-MnO exhibited the greatest protective effect against salinity-induced damage (Ghassemi-Golezani et al., 2021). Similarly, Sheikhalipour et al. investigated the role of selenium-doped carbon nanoparticles (Se-CS NPs) in enhancing the salt tolerance of Momordica charantia. Treatment with Se-CS NPs significantly improved plant growth, photosynthetic performance, and antioxidant capacity under saline conditions, as evidenced by increased SPAD values, Fv/Fm ratios, and the activities of antioxidant enzymes, including POD, SOD, and CAT. In addition, Se-CS NPs contributed to the maintenance of ionic homeostasis by regulating the accumulation of Na^+^, K^+^, Ca²^+^, and Cl^-^ and induced the expression of stress-responsive genes associated with salinity tolerance (Sheikhalipour et al., 2023). Collectively, current evidence suggests that nanomaterials enhance plant resilience to salt stress through the integrated regulation of osmotic adjustment, ion transport, antioxidant defense, and stress-responsive signaling pathways. By promoting the accumulation of compatible osmolytes, maintaining ionic balance, and facilitating the scavenging of excessive ROS, nanomaterials mitigate the physiological and biochemical damage caused by salinity. These protective effects contribute to improved plant growth, survival, and productivity under saline conditions, highlighting the considerable potential of nanomaterials for sustainable crop production in salt-affected agricultural systems.

##### 3.1.3.4 The role of nanomaterials under heavy metals stress

Heavy metal contamination represents a major environmental threat to plant growth and agricultural productivity. Heavy metals can enter plants through contaminated soil and water, where they disrupt physiological and metabolic processes, inhibit root and shoot development, and ultimately lead to growth retardation, chlorosis, and, in severe cases, plant death. At the cellular level, heavy metals impair chlorophyll biosynthesis and photosynthetic efficiency, disrupt membrane integrity, induce oxidative stress, and interfere with metabolic homeostasis, potentially triggering programmed cell death under prolonged exposure (Guo et al., 2023**;** Asgher et al., 2024**;** Panda et al., 2025).

Recent studies have demonstrated that nanomaterials can mitigate heavy metal-induced phytotoxicity through multiple physiological and molecular mechanisms. In a hydroponic study, Wang et al. investigated the effects of cadmium nanoparticles, copper nanoparticles (Cu NPs), and copper oxide nanoparticles (CuO NPs) on Brassica plants. The results showed that copper-based nanoparticles enhanced photosynthetic performance and biomass accumulation while significantly increasing the activities of antioxidant enzymes, including SOD, POD, and CAT. Notably, the combined Cd + Cu NP treatment exhibited an antagonistic effect on cadmium accumulation, reducing Cd concentrations in both roots and leaves. In contrast, the Cd + CuO NP treatment promoted cadmium accumulation in plant tissues. Furthermore, copper concentrations in aboveground tissues were negatively correlated with cadmium uptake, suggesting an important role of Cu NPs in regulating metal homeostasis under cadmium stress (Wang et al., 2022a). Similarly, Yan et al. demonstrated that both bulk silicon (Si) and silicon nanoparticles (Si NPs) enhanced tomato growth under cadmium stress, with principal component analysis indicating superior performance of Si NPs. Silicon nanoparticles effectively alleviated oxidative damage in both shoots and roots, whereas bulk Si primarily exerted protective effects in root tissues. Moreover, both treatments reduced cadmium concentrations in roots, shoots, and xylem sap by decreasing cadmium influx and extracellular uptake. At the molecular level, bulk Si regulated the expression of genes involved in cadmium uptake (*NRAMP2* and *LCT1*) and sequestration (*HMA3*), whereas Si NPs primarily reduced *NRAMP2* expression, thereby limiting cadmium accumulation within plant tissues (Yan et al., 2023). Collectively, current evidence suggests that nanomaterials can effectively enhance plant tolerance to heavy metal stress by improving antioxidant capacity, maintaining photosynthetic activity, regulating metal uptake and translocation, and modulating stress-responsive gene expression. These protective effects reduce heavy metal accumulation and oxidative damage, thereby improving plant growth, physiological performance, and crop quality under contaminated conditions.

## 4 Interactions between phytohormones and nanomaterials

Phytohormones and nanomaterials constitute two interconnected regulatory components that collectively influence plant growth, development, and stress adaptation. Increasing evidence indicates that nanomaterials not only exert direct effects on plant physiological and biochemical processes but also function as modulators of phytohormone biosynthesis, transport, signaling, and metabolism. Through these interactions, nanomaterials can reshape endogenous hormonal homeostasis and thereby regulate plant responses to adverse environmental conditions (Tripathi et al., 2025**;** Kandhol et al., 2023**;** Polyakov et al., 2023).

The relationship between nanomaterials and phytohormones is characterized by extensive signaling crosstalk. Upon exposure to nanomaterials, plants frequently exhibit alterations in the levels of key stress-related hormones, including abscisic acid, ethylene, jasmonic acid, salicylic acid, auxin, gibberellins, and cytokinins (Tripathi et al., 2022**;** Bhattacharya et al., 2023). These hormonal changes are often associated with nanomaterials-induced modulation of ROS, calcium signaling, and mitogen-activated protein kinase (MAPK) cascades, forming an integrated regulatory network that coordinates stress perception and adaptive responses. In turn, phytohormones can influence nanoparticle uptake, translocation, and physiological activity by regulating root architecture, membrane transport processes, and cellular redox homeostasis. Importantly, numerous studies have demonstrated a synergistic relationship between nanomaterials and phytohormonal pathways in enhancing plant tolerance to abiotic stress (Li et al., 2026**;** Ding et al., 2025). Nanomaterials can amplify hormone-mediated responses by promoting abscisic acid-dependent stomatal regulation, improving auxin-regulated root development, enhancing jasmonic acid- and salicylic acid-mediated defense responses, and maintaining cytokinin- and gibberellin-associated growth processes under stress conditions. Through the coordinated regulation of antioxidant defense systems, osmotic adjustment, ion homeostasis, photosynthetic performance, and stress-responsive gene expression, the interaction between nanomaterials and phytohormones enables plants to optimize resource allocation and maintain growth under adverse environments (Sreekanth et al., 2026**;** Jardim-Messeder et al., 2025).

In summary, current evidence suggests that the beneficial effects of nanomaterials on plant stress tolerance are mediated not only through their physicochemical properties but also through their ability to modulate complex phytohormonal signaling networks. A deeper understanding of the molecular mechanisms underlying nanomaterials–phytohormone interactions will facilitate the development of next-generation nanotechnologies for sustainable crop improvement and climate-resilient agriculture.

## 5 Discussion

Plants have evolved sophisticated signaling networks to perceive and respond to abiotic stresses. In addition to classical signaling molecules such as Ca²^+^ and ROS, phytohormones serve as central regulators that initiate and coordinate stress-responsive signaling cascades upon environmental stimulation (Khan et al., 2023b**;** Wang et al., 2022b). Early stress-induced fluctuations in the levels of abscisic acid, ethylene, gibberellins, jasmonic acid, and salicylic acid reprogram metabolic activities and developmental processes, thereby enabling plants to adapt to adverse conditions. The dynamic crosstalk among hormonal signaling pathways integrates diverse environmental cues and orchestrates coordinated physiological, biochemical, and molecular responses that enhance plant resilience under abiotic stress.

Extensive evidence has demonstrated that exogenous phytohormone application can effectively mitigate the detrimental effects of abiotic stresses in crops. Current application strategies generally include pre-sowing seed priming, foliar treatment during vegetative growth, and post-flowering application to delay senescence and maintain productivity under stress conditions (**Table 1**). Through the modulation of stress-responsive pathways, exogenous phytohormones promote stress adaptation while simultaneously regulating plant growth and development.

**Table 1:** The effects of exogenously applying plant hormones involved in plant responses to abiotic stress under abiotic stress conditions.

Phytohormone	Abiotic stresses	Treatment	Species	Application method	Plant Response	Reference
		type				
Abscisic acid	Salt stress	Pre-sowing seed treatment	Oryza sativa	Seed pre-treatment	Physiological responses: Reduced Na^+^ uptake and root-to-shoot transport; decreased apoplastic bypass flow; improved ionic homeostasis; enhanced growth under salt stress Molecular/Mechanistic responses: ABA- and Si-mediated regulation of Na^+^ transport pathways; restriction of Na^+^ entry into shoots; maintenance of ion balance Stress outcome: Enhanced salinity tolerance and reduced salt toxicity	(Gurmani et al., 2013)
Abscisic acid	Drought stress	Foliar distribution during vegetative growth and post-anthesis application to delay senescence.	Triticum aestivum	At shoot enlargement and anthesis	Physiological responses: Improved plant water status; enhanced water-use efficiency; regulation of stomatal conductance; reduced transpirational water loss Molecular responses: ABA-mediated drought-response signaling Growth and agronomic responses: Improved plant performance under water deficit; increased grain yield Stress outcome: Enhanced drought tolerance and drought resilience	(Travaglia et al., 2010)
Ethylene	High temperature stress	Foliar distribution during vegetative growth	Triticum aestivum	Sprayed once before heat stress occurrence	Physiological responses: Increased ethylene production; accelerated grain maturation; shortened grain-filling period; increased kernel abortion Biochemical responses: Heat stress-induced elevation of ethylene biosynthesis Molecular responses: Activation of ethylene-mediated heat stress signaling pathways Growth and yield responses: Reduced grain development; decreased kernel set; yield loss in heat-sensitive cultivars Stress outcome: Increased heat sensitivity and reduced reproductive performance	(Hays et al., 2007)
Gibberellins	Salt stress	Foliar distribution during vegetative growth	Zea mays	Weekly from 10 till 45 DAG	Physiological responses: Increased plant height; enhanced shoot and root growth; improved biomass accumulation; enhanced salt tolerance Biochemical responses: Increased SOD, CAT, and POD activities; enhanced ROS scavenging; reduced oxidative damage Nutritional responses: Improved K^+^ and Ca²^+^ uptake; reduced Na^+^ toxicity; improved ionic balance Stress outcome: Enhanced salinity tolerance and growth performance	(Tuna et al., 2008; Kaya et al., 2006)
Gibberellins	Drought stress	Foliar distribution during vegetative growth	Oryza sativa	At panicle initiation	Physiological responses: Improved plant water status; maintenance of photosynthetic activity; enhanced drought tolerance Growth responses: Improved vegetative growth and maintenance of growth under water deficit Source–sink regulation: Enhanced assimilate translocation and source–sink relationships Yield responses: Improved grain filling; increased grain yield and yield-related traits Stress outcome: Enhanced tolerance to water deficit stress	(Khan et al., 2016)
Salicylic acid	Salt stress	Pre-sowing seed treatment	Hordeum vulgare	Seed pre-treatment	Physiological responses Improved seed germination; enhanced root and shoot growth; increased seedling vigor; improved salt tolerance; Maintenance of chlorophyll content; alleviation of salt-induced chlorophyll degradation; improved photosynthetic capacity Biochemical responses: Enhanced osmotic adjustment; accumulation of compatible solutes; improved cellular water balance Growth responses: Promotion of seedling growth and biomass accumulation under salinity Stress outcome: Increased salinity tolerance and improved adaptation to salt stress	(El-Tayeb, 2005)
Salicylic acid	Salt stress	Foliar distribution during vegetative growth	Zea mays	Leaf spraying at 40 DAS	Physiological responses: Improved plant growth; increased biomass accumulation; enhanced salt tolerance Photosynthetic responses: Increased chlorophyll content; improved photosynthetic performance Biochemical responses: Enhanced antioxidant defense; increased proline accumulation; improved osmotic adjustment Growth responses: Increased plant height and overall vigor Stress outcome: Reduced salt-induced damage and enhanced salinity tolerance	(Fahad and Bano, 2012)
Salicylic acid	Salt stress	--	Zea mays	Incorporated into the soil (pot trial)	Physiological responses: Improved mineral nutrition; alleviated salt-induced growth inhibition; enhanced salinity tolerance Photosynthetic responses: Indirect improvement through maintenance of physiological status Biochemical responses: Reduced lipid peroxidation (lower MDA); decreased oxidative damage; enhanced antioxidant protection Growth responses: Improved plant growth and vigor under salinity Stress outcome: Enhanced salinity tolerance and reduced oxidative stress	(Gunes et al., 2007)
Cytokinin	Drought and high temperature stress	Pre-sowing seed treatment	Triticum aestivum	Seed pre-treatment	Physiological responses: Improved leaf water status; maintained physiological activity; enhanced tolerance to combined drought and heat stress; Improved photosynthetic performance; maintenance of chlorophyll/photosynthetic capacity Biochemical responses: Enhanced antioxidant defense; improved ROS scavenging; reduced oxidative damage Growth responses: Increased seedling growth and biomass; reduced stress-induced growth inhibition Developmental responses: Delayed senescence; sustained metabolic activity Stress outcome: Enhanced tolerance to combined drought and high-temperature stress	(Kumari et al., 2018)
Cytokinin	Drought stress	Post-anthesis application to delay senescence	Triticum aestivum	Sprayed from 9 DPA	Physiological responses: Enhanced remobilization of stored carbon reserves; improved assimilate transport to grains; maintenance of grain filling under drought; Reduced current photosynthesis under drought, compensated by reserve remobilization Biochemical responses: Increased endogenous ABA levels; altered cytokinin concentrations Developmental responses: Regulation of leaf senescence; optimization of source–sink relationships Molecular/Hormonal responses: ABA promoted assimilate translocation and sink activity; cytokinins regulated senescence processes Yield responses: Improved grain filling and maintenance of grain weight under water stress Stress outcome: Enhanced adaptation to drought during grain filling	(Yang et al., 2003)
Cytokinin	High temperature stress	Post-anthesis application to delay senescence	Triticum aestivum	Sprayed daily for 3 days after anthesis	Physiological responses Delayed leaf senescence; enhanced stay-green traits; improved heat tolerance; Increased chlorophyll retention; maintained photosynthetic activity and functional leaf area Biochemical responses: Reduced oxidative damage; enhanced antioxidant protection Hormonal responses: Cytokinin-mediated regulation of senescence processes Growth and yield responses: Improved grain filling; increased grain weight and grain yield Stress outcome: Enhanced tolerance to heat stress and maintenance of productivity	(Yang et al., 2016)
Cytokinin	Waterlogging stress	Foliar distribution during vegetative growth	Zea mays	Sprayed the day after waterlogging stress	Physiological responses: Delayed leaf senescence; enhanced recovery after waterlogging; improved leaf area duration; Increased chlorophyll content; maintained photosynthetic rate; prolonged photosynthetic activity Biochemical responses: Enhanced antioxidant defense; reduced oxidative damage Growth responses: Increased dry matter accumulation; improved biomass production Source–sink responses: Enhanced assimilate accumulation and translocation to grains Yield responses: Increased kernel number, grain weight, and grain yield Stress outcome: Improved tolerance and recovery from waterlogging stress	(Ren et al., 2016)
Cytokinin	Waterlogging stress	Foliar distribution during vegetative growth	Zea mays	Sprayed on waterlogged plants	Physiological responses: Enhanced shoot and root growth; increased biomass accumulation; alleviated growth inhibition caused by salinity and waterlogging Hormonal responses: Modulated endogenous plant growth regulator levels; restored hormonal balance under stress Biochemical responses: Maintained metabolic activity; delayed stress-induced senescence Growth responses: Improved plant vigor and growth under stress conditions Stress outcome: Enhanced tolerance to salinity and waterlogging stress	(Younis et al., 2003)

Conventional genetic engineering approaches have also contributed substantially to improving crop tolerance to abiotic stresses. However, their practical implementation is often limited by lengthy breeding cycles, technical complexity, regulatory constraints, and dependence on tissue culture-based regeneration systems. In this context, nanotechnology has emerged as a promising complementary strategy for enhancing crop resilience. Owing to their nanoscale dimensions and unique physicochemical properties, nanomaterials can efficiently penetrate plant tissues and modulate a wide range of physiological, biochemical, and molecular processes under stress conditions. As a versatile and highly adaptable platform, nanotechnology offers significant potential for improving plant performance and stress tolerance across diverse agricultural environments (**Table 2**).

**Table 2:** Mechanisms by which nanoparticles enhance plants stress tolerance under abiotic stress conditions.

Nanoparticles	Abiotic stresses	Usage	Crop species	Impact	Reference
AgNPs	Drought stress	Seed-induced	*Oryza sativa*	AgNPs can enhance water absorption, increase α-amylase activity, and promote ROS signaling and aquaporin expression	(Mahakham et al., 2017)
CaO-NPs	Drought stress	Seed-induced	*Brassica napus*	CaO-NPs can significantly increase rapeseed yield under drought conditions by increasing antioxidant enzyme activity, reducing ROS damage.	(Mazhar et al., 2022)
SeNPs	Drought stress	Leaf application	*Triticum aestivum*	SeNPs can enhance antioxidant defense, protect photosynthesis, regulate osmotic balance, and activate the expression of stress-responsive genes.	(Omar et al., 2023)
SeNPs	Drought stress	Leaf application	*Punica granatum*	SeNPs can significantly alleviate the inhibitory effects of drought by enhancing the antioxidant system, improving plant water status, promoting osmotic regulation, and protecting photosynthesis.	(Zahedi et al., 2021)
ZnO-NPs	Drought stress	Seed-induced	*Zea mays*	ZnO-NPs can significantly alleviate drought stress by improving stomatal regulation, enhancing water use efficiency, protecting photosynthesis, and reprogramming carbohydrate metabolism	(Sun et al., 2021)
GO	Drought stress	Soil-root uptake	*Glycine max*	GO significantly enhances tolerance to drought stress by improving soil water-holding capacity, promoting root development, strengthening the antioxidant system, regulating ABA/JA/SA hormone signaling, and upregulating the expression of drought-related genes.	(Zhao et al., 2022a)
AgNPs	High temperature stress	Soil-root uptake	*Triticum aestivum*	AgNPs can increase the root-to-shoot ratio, reduce ROS levels, and enhance wheat heat tolerance.	(Iqbal et al., 2019)
TiO_2_-NPs	High temperature stress	Leaf application	*Sesamum indicum*	TiO2-NPs can significantly alleviate the damage caused by high-temperature stress to plants by enhancing photosynthesis, increasing antioxidant enzyme activity, promoting osmotic regulation, and increasing the accumulation of antioxidant metabolites such as phenolics and flavonoids.	(Mahmoud and Abdelhameed, 2023)
CTS-GB-NPs	Cold temperature stress	Fruit spread	*Prunus salicina*	CTS-GB-NPs can significantly alleviate cold damage and maintain fruit quality by forming a protective edible coating, enhancing the antioxidant system, promoting osmoregulation, and activating the metabolism of phenolic compounds and anthocyanins,	(Mahmoudi et al., 2022)
TiO_2_-NPs	Cold temperature stress	Seed-induced	*Cicer arietinum*	TiO2-NPs can alleviate the inhibitory effects of low temperatures on photosynthesis by improving stomatal conductance, increasing Rubisco activity and ATP production, protecting photosystem II, and reducing low-temperature-induced non-stomatal metabolic constraints.	(Hasanpour et al., 2015)
CeO_2_-NPs	Salt stress	Root uptake	*Oryza sativa*	CeO₂-NPs can alleviate the toxic effects of salt-compound stress by scavenging ROS, enhancing antioxidant enzyme activity, and maintaining Na⁺/K⁺ ion homeostasis through its “nanoenzyme” properties.	(Wang et al., 2019b)
ZnO-NPs	Salt stress	Leaf application	*Vicia faba*	ZnO-NPs can significantly alleviate the inhibitory effects of salt stress on growth and yield by improving Na⁺/K⁺ ion balance, enhancing antioxidant enzyme activity, promoting osmotic regulation, and maintaining the stability of the photosynthetic system.	(Mogazy and Hanafy, 2022)
ZnO-NPs	Salt stress	Leaf application	*Triticum aestivum*	ZnO can significantly enhance wheat tolerance to salt stress by promoting the accumulation of proline and soluble sugars, increasing the uptake of K⁺ and Ca²⁺, and enhancing overall metabolic capacity.	(Lalarukh et al., 2022)
CeO_2_-NPs	Salt stress	Leaf injection	*Gossypium hirsutum Linn*	CeO₂ can eliminate ROS, reduce membrane depolarization, inhibit GORK-mediated K⁺ efflux, and enhance Na⁺ efflux and vacuolar sequestration through its “nanocatalytic” activity, thereby maintaining cytoplasmic K⁺/Na⁺ homeostasis and improving tolerance to salt stress.	(Liu et al., 2021)
CeO_2_-NPs	Salt stress	Soil-root uptake	*Brassica napus*	CeO₂ weakens the ion barrier function of plant roots and exacerbates salt stress damage by inhibiting the formation of the Casparian strip and cork layer in rapeseed roots, enhancing Na⁺ bypass transport through the plastid membrane, and promoting Na⁺ accumulation.	(Rossi et al., 2017)
FeSO_4_-NPs	Salt stress	Leaf application	*Helianthus annuus*	FeSO₄-NPs can significantly alleviate the damage caused by salt stress by enhancing the accumulation of proline and soluble sugars, increasing the activity of antioxidant enzymes such as SOD, POD, and CAT, reducing membrane lipid peroxidation, and improving photosynthesis and ion balance.	(Torabian et al., 2018)
SeNPs	Heavy Metals Stress	Root uptake	*Oryza sativa*	SeNPs are most effective at reducing arsenic accumulation and toxicity by inhibiting the transport of arsenic to the aboveground parts of the plant and enhancing antioxidant defenses.	(Wang et al., 2021b)
CeO_2_-NPs	Heavy Metals Stress	Root uptake	*Oryza sativa*	Through its nanozyme activity, CeO2-NPs can reduce ROS accumulation, enhance the antioxidant system, decrease Cd absorption and transport, and improve the K⁺/Na⁺ ion balance, thereby significantly alleviating the toxic effects of combined Cd and salt stress on rice seedlings.	(Wang et al., 2019b)
ZnO-NPs	Heavy Metals Stress	Leaf application	*Oryza sativa*	The combined application of ZnO-NPs biochar and ZnO nanoparticles can significantly reduce Cd accumulation in rice and alleviate Cd toxicity by immobilizing soil Cd, reducing Cd bioavailability, enhancing the plant’s antioxidant system, improving photosynthesis, and utilizing the Zn-Cd competitive uptake mechanism.	(Ali et al., 2021)
Fe_3_O_4_-NPs	Heavy Metals Stress	Root uptake	*Oryza sativa*	Fe3O4-NPs synthesized via a green method using rubber tree bark can significantly alleviate the toxic effects of combined Cd and salt stress on rice by efficiently adsorbing Cd²⁺ and Na⁺, reducing their bioavailability, enhancing the antioxidant system, and protecting root tissue structure.	(Sebastian et al., 2019)
SiNPs	Heavy Metals Stress	Root uptake	*Oryza sativa*	SiNPs forms hemicellulose-bound Si by interacting with hemicellulose in rice cell walls, thereby enhancing the cell walls’ ability to bind and adsorb Cd²⁺. This creates a metal barrier at the cell wall level, significantly inhibiting Cd uptake into cells and alleviating cadmium toxicity in rice.	(Ma et al., 2015)
Fe_3_O_4_-NP	Heavy Metals Stress	Seed-induced	*Phaseolus vulgaris*	The combined application of Fe₃O₄-NPs and Si-NPs can synergistically alleviate the toxic effects of Cd stress on common beans by maintaining the homeostasis of mineral elements such as K⁺, reducing Cd accumulation, enhancing the antioxidant system, promoting polyamine and proline metabolism, and improving photosynthesis.	(Koleva et al., 2022)
SiNPs	Heavy Metals Stress	Root uptake	*Momordica charantia*	SiNPs can significantly alleviate the toxic effects of Cd on bitter gourd seedlings by enhancing the activity of antioxidant enzymes such as SOD, POD, and CAT; reducing the accumulation of ROS and MDA; promoting proline synthesis; improving photosynthesis; and inhibiting Cd uptake and transport.	(Sun et al., 2023)
CuNPs	Heavy Metals Stress	Root uptake	*Triticum aestivum*	CuNPs can significantly mitigate the toxic effects of chromium on wheat by reducing its bioavailability, decreasing chromium accumulation in plants, enhancing antioxidant systems such as SOD, POD, and CAT, and alleviating ROS-mediated oxidative damage.	(Noman et al., 2020)
AgNPs	Waterlogging stress	Root uptake	*Glycine max*	AgNPs can significantly enhance soybean tolerance to waterlogging stress by boosting energy metabolism, increasing antioxidant capacity, improving protein homeostasis, and regulating the expression of root and cell wall-related proteins.	(Hashimoto et al., 2020)
Graphene-NPs	Salt stress	Root fertilization	*Medicago sativa*	Graphene-NPs can enhance photosystem function, maintain Na⁺/K⁺ homeostasis, and increase the activity of antioxidant enzymes such as SOD, CAT, POD, and APX by regulating the expression of genes related to photosynthesis, antioxidant defense, and ion transport, thereby significantly alleviating growth inhibition and oxidative damage in alfalfa under salt stress.	(Chen et al., 2021b)
Graphene-NPs	Alkali stress	Root fertilization	*Medicago sativa*	Graphene-NPs can enhance photosystem function, maintain Na⁺/K⁺ homeostasis, and increase the activity of antioxidant enzymes such as SOD, CAT, POD, and APX by regulating the expression of genes related to photosynthesis, antioxidant defense, and ion transport, thereby significantly alleviating growth inhibition and oxidative damage in alfalfa under alkaline stress.	(Chen et al., 2021b)

Despite the substantial progress achieved in laboratory and greenhouse studies, the translation of nanotechnology-based strategies into practical agricultural applications remains at a relatively early stage. Most investigations assessing the effects of nanomaterials on plant growth and abiotic stress tolerance have been conducted under controlled conditions, which often fail to capture the complexity and heterogeneity of real-world agricultural systems. Environmental factors, including soil physicochemical properties, climatic variability, microbial community dynamics, irrigation regimes, and crop management practices, can profoundly influence the fate, bioavailability, transport, and efficacy of NMs. Consequently, multi-location and long-term field trials across diverse agroecosystems are urgently needed to validate experimental findings and evaluate the agronomic performance and stability of nano-enabled technologies under realistic cultivation conditions. From a commercialization perspective, nanotechnology holds considerable promise for the development of next-generation fertilizers, biostimulants, agrochemicals, and crop protection products. Nanoformulations can enhance the stability, bioavailability, targeted delivery, and controlled-release characteristics of active ingredients, thereby improving resource-use efficiency while reducing chemical inputs and environmental losses. Nevertheless, several challenges continue to hinder large-scale commercialization, including high production costs, manufacturing complexity, limited scalability, and inconsistencies in nanoparticle physicochemical properties among production batches. Overcoming these technical and economic barriers will be essential for the successful deployment of nano-enabled products in modern agriculture. In parallel, regulatory frameworks governing agricultural nanomaterials remain fragmented and underdeveloped in many parts of the world. Existing regulations are frequently adapted from those established for conventional chemicals and may not adequately address the unique physicochemical characteristics, environmental behavior, and biological interactions of nanoscale materials. Therefore, the establishment of harmonized international guidelines for the evaluation, registration, labeling, and risk assessment of nano-enabled agricultural products is urgently required. In particular, standardized methodologies for nanoparticle characterization, exposure assessment, environmental monitoring, and safety evaluation are critical for supporting evidence-based regulatory decisions and facilitating the responsible commercialization of agricultural nanotechnologies.

## 6 The future prospects and conclusions

Although substantial progress has been made in elucidating the roles of nanomaterials in enhancing plant tolerance to abiotic stresses, future research is expected to move beyond conventional nanoparticle applications toward the development of integrated, intelligent, and sustainable agricultural systems. In this context, several emerging research directions deserve particular attention.

One promising avenue is the application of nanotechnology in precision agriculture. The development of nano-enabled sensing platforms capable of real-time monitoring of soil moisture, nutrient availability, phytohormone dynamics, and stress-associated biomarkers will facilitate early stress detection and enable site-specific crop management. Such technologies have the potential to substantially improve resource-use efficiency while reducing excessive inputs of fertilizers and agrochemicals, thereby promoting more sustainable agricultural practices.

Another rapidly evolving frontier is the development of smart nanomaterials. Unlike conventional nanoparticles, stimulus-responsive nanomaterials can be engineered to release nutrients, phytohormones, or other bioactive compounds in response to specific environmental cues, including drought, salinity, temperature fluctuations, pH changes, and oxidative stress. These intelligent delivery systems offer unprecedented opportunities for precise spatiotemporal control of active ingredient release, thereby maximizing efficacy while minimizing environmental impacts and off-target effects.

The integration of artificial intelligence with nano-enabled agricultural technologies further expands the potential of data-driven crop management. By combining nanosensors, remote sensing platforms, internet of things networks, and machine-learning algorithms, large-scale datasets can be analyzed to predict stress occurrence, optimize nanoparticle application regimes, and support real-time agronomic decision-making. Such artificial intelligence-assisted systems are expected to improve the precision, efficiency, and scalability of nanotechnology-based agricultural interventions.

Equally important is the convergence of nanotechnology with advanced breeding technologies. Nanoparticles have emerged as promising delivery vehicles for CRISPR/Cas9-based genome-editing systems, RNA molecules, and other biomacromolecules, providing potential alternatives to conventional transformation approaches that rely heavily on tissue culture. The integration of nanotechnology with genome editing, molecular breeding, and synthetic biology may accelerate the development of next-generation crop varieties with enhanced resilience to multiple abiotic stresses and changing climatic conditions.

In addition, increasing evidence indicates that nanomaterials can modulate phytohormone biosynthesis, transport, metabolism, and signaling. Future studies should therefore focus on elucidating the molecular mechanisms underlying nanomaterials–phytohormone interactions and identifying potential synergistic effects between nanomaterials and hormone-mediated stress responses. A deeper understanding of these regulatory networks will facilitate the rational design of next-generation nanoplatforms capable of precisely manipulating endogenous signaling pathways to enhance plant adaptation to environmental challenges.

This review highlights the pivotal roles of phytohormones and nanomaterials in enhancing plant tolerance to abiotic stresses and provides a comprehensive synthesis of the underlying physiological, biochemical, and molecular mechanisms. A key finding emerging from this review is that the extensive crosstalk between nanomaterials-mediated responses and phytohormone signaling pathways constitutes a central regulatory network governing plant adaptation to environmental stresses. Increasing evidence indicates that nanomaterials can modulate phytohormone biosynthesis, transport, metabolism, and signaling, thereby coordinating antioxidant defense, osmotic adjustment, ion homeostasis, photosynthetic performance, and stress-responsive gene expression. Through these integrated regulatory processes, plants are better equipped to withstand drought, salinity, temperature extremes, and heavy metal stress. Beyond summarizing current mechanistic insights, this review also explores emerging research frontiers, including smart nanomaterials, nano-enabled precision agriculture, artificial intelligence-assisted crop management, advanced breeding technologies, commercialization prospects, regulatory frameworks, and environmental safety considerations. By integrating these rapidly evolving topics into a unified perspective, this review provides a roadmap for future research and highlights the potential of nanotechnology to contribute to sustainable and climate-resilient agricultural systems.

In conclusion, the future of agricultural nanotechnology will likely be shaped by the convergence of smart nanomaterials, phytohormone-regulated signaling networks, precision agriculture, artificial intelligence, and advanced breeding technologies. Such interdisciplinary integration has the potential to accelerate the development of climate-resilient crops, enhance resource-use efficiency, and promote sustainable agricultural production systems capable of addressing the growing challenges of climate change, environmental degradation, and global food insecurity.
